# Eukaryote-like Ser/Thr protein kinase PrkA modulates sporulation via regulating the transcriptional factor σ^K^ in *Bacillus subtilis*

**DOI:** 10.3389/fmicb.2015.00382

**Published:** 2015-04-30

**Authors:** Jinyuan Yan, Wei Zou, Juan Fang, Xiaowei Huang, Feng Gao, Zeying He, Keqin Zhang, Ninghui Zhao

**Affiliations:** ^1^Laboratory for Conservation and Utilization of Bio-Resources, and Key Laboratory for Microbial Resources of the Ministry of Education, Yunnan UniversityKunming, China; ^2^Department of Neurosurgery, The Second Affiliated Hospital of Kunming Medical CollegeKunming, China

**Keywords:** PrkA, serine/threonine protein kinase, sporulation, *B. subtilis*, the transcription factor σ^K^, transcriptional regulation

## Abstract

Protein kinase A (PrkA), also known as AMP-activated protein kinase, functions as a serine/threonine protein kinase (STPK), has been shown to be involved in a variety of important biologic processes, including pathogenesis of many important diseases in mammals. However, the biological functions of PrkA are less known in prokaryote cells. Here, we explored the function of PrkA as well as its underlying molecular mechanisms using the model bacterium *Bacillus subtilis*168. When PrkA is inhibited by 9-β-D-arabinofuranosyladenine (ara-A) in the wild type strain or deleted in the Δ*prkA* mutant strain, we observed sporulation defects in *B. subtilis* 168, suggesting that PrkA functions as a sporulation-related protein. Transcriptional analysis using the *lacZ* reporter gene demonstrated that deletion of *prkA* significantly reduced the expression of the transcriptional factor σ^K^ and its downstream genes. Complementation of *sigK* gene in *prkA* knockout mutant partially rescued the phenotype of Δ*prkA*, further supporting the hypothesis that the decreased σ^K^ expression should be one of the reasons for the sporulation defect resulting from *prkA* disruption. Finally, our data confirmed that Hpr (ScoC) negatively controlled the expression of transcriptional factor σ^K^, and thus PrkA accelerated sporulation and the expression of σ^K^ by suppression of Hpr (ScoC). Taken together, our study discovered a novel function of the eukaryotic-like STPK PrkA in spore development as well as its underlying molecular mechanism in *B. subtilis*.

## Introduction

Protein phosphorylation is the principal mechanism by which extracellular signals are translated into cellular responses. A variety of protein kinases are responsible for the reversible phosphorylation at specific amino acid residues, including serine/threonine protein kinases (STPKs) that phosphorylate serine or threonine of the target proteins. Contrary to the signaling pathways predominantly carried out by STPKs in eukaryotes, two-component systems, consisting of His-kinase sensors and their associated response regulators, are the most common signal transduction system in prokaryotes. Therefore, serine, threonine, and tyrosine kinases had been previously thought to be unique to eukaryotes until phosphorylation at Ser residues was identified in bacteria (Deutscher and Saier, 1988; Reizer et al., 1993). Recent data from genomic sequencing has further illustrated that the eukaryote-like STPKs exist widely in bacteria, suggesting that this well-characterized protein phosphorylation is also distributed in prokaryotes along with two-component systems (Bakal and Davies, 2000). For example, the genome of *Mycobacterium tuberculosis* contains 11 STPKs (Cole et al., 1998; Av-Gay and Everett, 2000). These STPKs are involved in a variety of processes such as development, cell growth, stress responses, primary and secondary metabolism, biofilm formation, antibiotic resistance, and virulence (Cozzone, 2005; Kristich et al., 2007; Wehenkel et al., 2008; Molle and Kremer, 2010; Ohlsen and Donat, 2010).

The AMP-activated protein kinase (AMPK), also called protein kinase A (PrkA), is an important type of STPK. In eukaryotic cells, AMPK is a highly conserved heterotrimeric protein consisting of a catalytic α subunit and regulatory β and γ subunits, and it is regulated by the intracellular ratio of AMP to ATP (Osler and Zierath, 2008). AMPK is activated under conditions of low cellular ATP and high cellular AMP. Therefore, AMPK functions as an energy sensor in cells, and a likely metabolic master switch to coordinate global metabolic response involving cellular uptake of glucose, glycogen synthesis and decomposition, β-oxidation of fatty acids, and mitochondrial biogenesis. At the meantime, AMPK also participates in pathogenesis of important diseases such as ischemic heart, diabetes, cancer, and even viral infection. As a result, it has become a research focus in recent years (Spasic et al., 2009; Kuznetsov et al., 2011).

Comparing to the well-described functions of AMPK in eukaryotic cells, few investigations of AMPK have been reported in prokaryotes. The first gene encoding a prokaryotic PrkA was cloned in *Bacillus subtilis*. Though the sequence of this PrkA exhibited distant homology to eukaryotic proteins, it phosphorylated a 60-kDa target protein at a Ser residue. However, the biological functions of PrkA remained unclear because no phenotypic change was obtained when the *prkA* gene was deleted (Fischer et al., 1996). Later, it has been demonstrated that the transcription of *prkA* was regulated by the spore-specific sigma factor σσ^E^ (Eichenberger et al., 2003; Steil et al., 2005). Its role was also involved in spore formation because of its localization of spore coat and the decreased sporulation efficiency in *prkA* knockout mutant (Eichenberger et al., 2003). However, how PrkA works requires further elucidation. Additionally, another prokaryotic STPK PrkA was identified in a Gram-positive rod-shaped bacterium *Listeria monocytogenes* (Lima et al., 2011). Analysis of its potential interaction partners through proteomic approaches suggested that the signal transduction pathways mediated by PrkA in *L. monocytogenes* may affect a variety of fundamental functions, such as protein synthesis, cell wall metabolism, and carbohydrates metabolism (Lima et al., 2011). Since the relatively poor understanding on prokaryotic PrkA, we investigate STPK PrkA in *B. subtilis* strain 168 and demonstrate its roles in sporulation as well as the underlying molecular mechanism.

## Materials and Methods

### Bacterial Strains, Plasmids, and Media

The strains of *B. subtilis* and *Escherichia coli* as well as the plasmids used in this study are listed in **Table 1**. All *B. subtilis* strains were derivatives of *B. subtilis* 168, and those constructed in this work were prepared via transformation with plasmid DNA, confirmed by PCR analysis, and sequenced to ensure that the targeted changes were made. The oligonucleotides primers used for PCR amplification in this study are listed in **Table 2**>.

**Table 1:** Bacterial strains and plasmids used in this study.

Strain or plasmid	Genotype/description	Source or reference
**Strains**
*Bacillus subtilis* 168	Wild type (WT)	From Bacillus Genetic Stock Center
PRKA5E	*prkA*::cat	This work(pPRKA5E, 168)
SPOIVBBS	*spoIVB* (σ^F^)::pDG1728, reporter, control	This work(pSPOIVB, 168)
GERDBS	*gerD* (σ^G^)::pDG1728, reporter, control	This work(pGERD, 168)
GEREBS	*gerE* (σ^K^)::pDG1728, reporter, control	This work(pGERE,168)
SPOIVBPRKA5E	*spoIVB* (σ^F^)::pDG1728, reporter	This work(pSPOIVB, PRKA5E)
GERDPRKA5E	*gerD* (σ^G^)::pDG1728, reporter	This work(pGERD, PRKA5E)
GEREPRKA5E	*gerE* (σ^K^)::pDG1728, reporter	This work(pGERE, PRKA5E)
SIGKPRKA5E	*spoIVCB and spoIIIC*::pDG148	This work(pSIGKPRKA5E, PRKA5E)
PDG148PRK5E	pDG148, control	This work(pDG148, PRKA5E)
RSIGKBS	*sigK*::pDG1728, reporter, control	This work(pRSIGK, 168)
RSIGEBS	*sigE*::pDG1728, reporter, control	This work(pRSIGE, 168)
RHPRBS	*hpr*::pDG1728, reporter, control	This work(pRHPR, 168)
RGLNRBS	*glnR*::pDG1728, reporter, control	This work(pRGLNR, 168)
RSIGDBS	*sigD*::pDG1728, reporter, control	This work(pRSIGD,168)
RSIGKPRKA5E	*sigK*::pDG1728, reporter	This work(pRSIGK, PRKA5E)
RSIGEPRKA5E	*sigE*::pDG1728, reporter	This work(pRSIGE, PRKA5E)
RHPRPRKA5E	*hpr*::pDG1728, reporter	This work(pRHPR, PRKA5E)
RGLNRPRKA5E	*glnR*::pDG1728, reporter	This work(pRGLNR, PRKA5E)
RSIGDPRKA5E	*sigD*::pDG1728, reporter	This work(pRSIGD, PRKA5E)
*Escherichia coli* DH5	*lac*ZYA-*arg*F, *end*A1, *rec*A1, *hsd*R17, *sup*E44λ-*thi*-1, *gyr*A96, *rel* A1, *pho*A	TaKaRa
**Plasmids**
pMD19-T	*Amp*	TaKaRa
pDG1728	*Bla*, *emr*, *spc*, *spoVG-lacZ*, *amyE*, P*spac*	Bacillus Genetic Stock Center
pDG148	*kanR*, *ampR*, *lacI*, *ph1R*, P*pen*, P*spac*	Bacillus Genetic Stock Center
pPRKA5E	*Amp*, *cat*, *prkA*	This work
pSPOIVB	*Amp*, *spc*, *spoIVB-lacZ*	This work
pGERD	*Amp*, *spc*, *gerD-lacZ*	This work
pGERE	*Amp*, *spc*, *gerE-lacZ*	This work
pSIGKPRKA5E	*kanR*, *ampR*, *sigK*	This work
pRSIGK	*Amp*, *spc*, *SigK-lacZ*	Synthesized by Shanghai General Co.
pRSIGE	*Amp*, *spc*, *SigE-lacZ*	Synthesized by Shanghai General Co.
pRHPR	*Amp*, *spc*, *Hpr-lacZ*	Synthesized by Shanghai General Co.
pRGLNR	*Amp*, *spc*, *GlnR-lacZ*	Synthesized by Shanghai General Co.
pRSIGD	*Amp*, *spc*, *SigD-lacZ*	Synthesized by Shanghai Generaly Co.

**Table 2:** The oligonucleotide primers used in this study.

Name	Sequence (5′–3′)	Function and Source
PRKA1F	GACAGCGGGATAGAGGAGA	pPRKA5E
PRKA1R	CCAACCCGTTCCATGTGCTCCAAT ACTCCCCGTCAAATCG	pPRKA5E
CATF	CGATTTGACGGGGAGTATTGGAGCAC ATGGAACGGGTTGG	pPRKA5E
CATR	ATATCGTCATATTCCTTGCTTCCGAGGCTCAACGTCAAT	pPRKA5E
PRKA2F	ATTGACGTTGAGCCTCGGAAGC AAGGAATATGACGATAT	pPRKA5E
PRKA2R	CCGACATATTTCAGCAGCTC	pPRKA5E
SPOIVBF	GAATTCATTTTTTTCGTGCACATCCA	pSPOIVB
SPOIVBR	GGATCCTCTCATTTGCGTTGGAATCA	pSPOIVB
GERDF	GAATTCATTCATCCCCTCAAAAATCG	pGERD
GERDR	GGATCCTAACAAAAAACAGCTCATCA	pGERD
GEREF	GAATTCACTAATTATCTTGTAAACGTCAC	pGERE
GERER	GGATCCACGGTTTTCTCACTGATAAA	pGERE
RTSPOIVBF	CTGGTGAATCTTTAGACTTACTG	Realtime-PCR
RTSPOIVBR	GATTCTGTATTTGCCTTCTCCTT	Realtime-PCR
RTSPOIVCBF	GATGAACATGCCAGAAACAT	Realtime-PCR
RTSPOIVCBR	AAGTCCTCTGCATCCTCAC	Realtime-PCR
RTSPOIIICF	GATAGATACGATCCAGCTCAAT	Realtime-PCR
RTSPOIIICR	AAACCGCCCGACAATCACTT	Realtime-PCR
RTGEREF	GATAAGACAACAAAGGAGATTGC	Realtime-PCR
RTGERER	CCTTTCACACCCAATTTCTGCAT	Realtime-PCR
RTMMGBF	GCGGACATTGTGATTGAGGC	Realtime-PCR
RTMMGBR	ATCGTATGAGGCGGGCAAAT	Realtime-PCR
RTSPOIIDF	ACGTACAACAACCAGCCGAT	Realtime-PCR
RTSPOIIDR	CCATGGGCTTTTGACGCT	Realtime-PCR
RTSPOVBF	TATCGAGTGTGCTGAGGGGA	Realtime-PCR
RTSPOVBR	CGAGTGAAATGCGGACAACC	Realtime-PCR
SIGK1F	AAGCTTATGGTGACAGGTGTTTTCG	pSIGKPRKA5E
SIGK1R	CATTACAAAAAGGGGGGGCATAC TCTTGAAGATAAA	pSIGKPRKA5E
SIGK2F	TTTATCTTCAAGAGTATGCCCCCC CTTTTTGTAATG	pSIGKPRKA5E
SIGK2R	GCATGCTTATTTCCCCTTCGCCTTCTTCCG	pSIGKPRKA5E

The strains were grown in Luria–Bertani medium, and sporulation was induced in 2x SG medium, a modified Schaeffer’s medium containing beef extract 0.3 g/L, peptone 0.5 g/L, MgSO_4_.7H_2_O 0.5 g/L, KCl 2.0 g/L, 10^-4^ M MnCl_2_, 10^-3^ M Ca(NO_3_)_2_, 10^-6^ M FeSO_4_. 7H_2_O and 0.1% glucose (Leighton and Doi, 1971). Antibiotics were used at the following concentrations: chloramphenicol 5 μg/ml, kanamycin 5 μg/ml, and spectinomycin 100 μg/ml.

### Genetic Manipulation

The double crossover of homologous recombination method was used to construct Δ*prkA* mutant (PRKA5E), Δ*hpr* mutant (HPR5E), and Δ*prkA*Δ*hpr* mutant (PRKA5E HPR5E) of *B. subtilis*. Two homologous fragments of the *prkA* gene, chloramphenicol resistant gene were amplified via PCR. The three fragments were linked by overlapping PCR and inserted into a pMD19-T vector to obtain plasmid pPRKA5E. *B. subtilis* 168 was transformed with plasmid pPRKA5E to generate the Δ*prkA* mutant strain PRKA5E. For the construction of Δ*hpr* mutant (HPR5E) and Δ*prkA*Δ*hpr* mutant (PRKA5E HPR5E), two homologous fragments of the *hpr* gene and erythromycin resistant gene were amplified via PCR, connected by overlap PCR, and inserted into a *pEASY*-T5 vector to obtain plasmid pHPR5E. The plasmid pHPR5E was finally transformed into *B. subtilis* 168 and Δ*prkA* mutant (PRKA5E) to obtain the Δ*hpr* mutant (HPR5E) and Δ*prkA*Δ*hpr* mutant (PRKA5E HPR5E), respectively.

The encoding gene of the transcriptional factor σ^K^ is formed due to a developmental DNA rearrangement of the two separate coding regions (*spoIVCB* and *spoIIIC*). To complement the expression of SigK, *spoIVCB*, and *spoIIIC* were amplified via PCR, linked together by overlapping PCR. The linked PCR product was digested with *Hind*III and *Sph*I at primer-incorporated restriction sites, and inserted into a *Hind*III/*Sph*I -digested pDG148 vector to obtain plasmid pSIGKPRKA5E. The recombinant plasmids pSIGKPRKA5E and the blank vector pDG148 were, respectively, transformed into the Δ*prkA* mutant strain to obtain the bacterial strain SIGKPRKA5E that complement the expressions of the *sigK* genes and the corresponding control strain PDG148PRK5E.

### Sporulation Assays

The bacterial strains were cultured in Luria-Bertani medium and shaken overnight at 37°C. The overnight cultures were transferred to 2x SG medium to induce sporulation. Spores were assayed at 12 , 24, and 36 h. The spore numbers per milliliter were measured by plating onto LB agar medium after applying a heat treatment (80°C for 15 min). The number of viable cells per milliliter was counted, both before and after the heat treatment, as total CFU on LB plates. Sporulation frequency is determined as the ratio of the number of spores per milliliter to the number of viable cells per milliliter (LeDeaux and Grossman, 1995). Data for each strain is from at least three independent experiments.

For the experiment of PrkA inhibitor, 250 μM 9-β-D-arabinofuranosyladenine (ara-A) from Sigma Co. was added into the 2x SG medium before adding overnight culture.

### β-Galactosidase Assays

Plasmid pDG1728 containing the *lacZ* gene, obtained from the Bacillus Genetic Stock Center (BGSC), was used for constructing reporter vectors for transformation. Segments of the reporter of *spoIVB*, *gerD*, and *gerE* genes were amplified via PCR, digested with *Eco*RI and *Bam*HI at the corresponding primer-incorporated restriction sites, and inserted into the *Eco*RI/*Bam*HI-digested pDG1728 vector to obtain plasmids pSPOIVB, pGERD, and pGERE.

To analyze the regulation of gene expression for the transcriptional factor σ^K^, five nested fragments that contained the truncated promoter region of σ^K^ were designed to fuse to pDG1728. Those five fragments above were sent to Shanghai Generay Biological Engineering Co. Ltd for synthesis. All the reporter plasmids, including pSIGK, pSIGE, pHPR, pGLINR, and pSIGD, were successfully constructed. Those constructed reporter plasmids were then transformed into wild type (WT) strain *B. subtilis* 168 and the mutant Δ*prkA*, respectively.

After the strains harboring *lacZ* fusions were cultured at 37°C in 2x SG medium to induce sporulation, they were assayed for β-galactosidase activity as previously described (Ferrari et al., 1988). β-galactosidase was assayed using o-nitrophenyl-β-D-galactopyranoside as the substrate and is reported in Miller units. Three repeats were performed at each time point.

### Real-Time PCR Assays

The cells of strains *B. subtilis* 168, PRKA5E, HPR5E, and PRKA5E HPR5E were grown for 12 h at 37°C in 2x SG medium, respectively, and 5 ml cultures were harvested by centrifugation. The total RNA was isolated using RNA extracting kit (Tiangen, China) following the treatment of DNaseI to avoid DNA contaminant. RNAclean Kit (BioTech, China) was then employed to further purify the total RNA. RNA concentration was determined by measuring absorbance at 260 nm using a UV spectrophotometer. After random-primed cDNAs were generated, qPCR analysis was performed with SYBR Green JumpStart Taq Ready Mix for qPCR kit (Sigma–Aldrich Co.) following manufacturer’s instructions. The partial sequence of 16S rRNA was used as an internal control. The PCR amplification used 40 cycles of 94°C for 30 s, 60°C for 30 s, 72°C for 40 s on ABI PRISM 7000 Real-Time PCR.

The real-time PCR experiments were repeated three times for each reaction using independent RNA sample.

### Statistical Analysis

All the data were expressed as the mean ± SD. Statistical comparisons were performed by a one-way analysis of variance (ANOVA) followed by Dunnett’s *t*-test.

## Results

### Effect of PrkA Inhibitor ara-A on Sporulation

**FIGURE 1:**
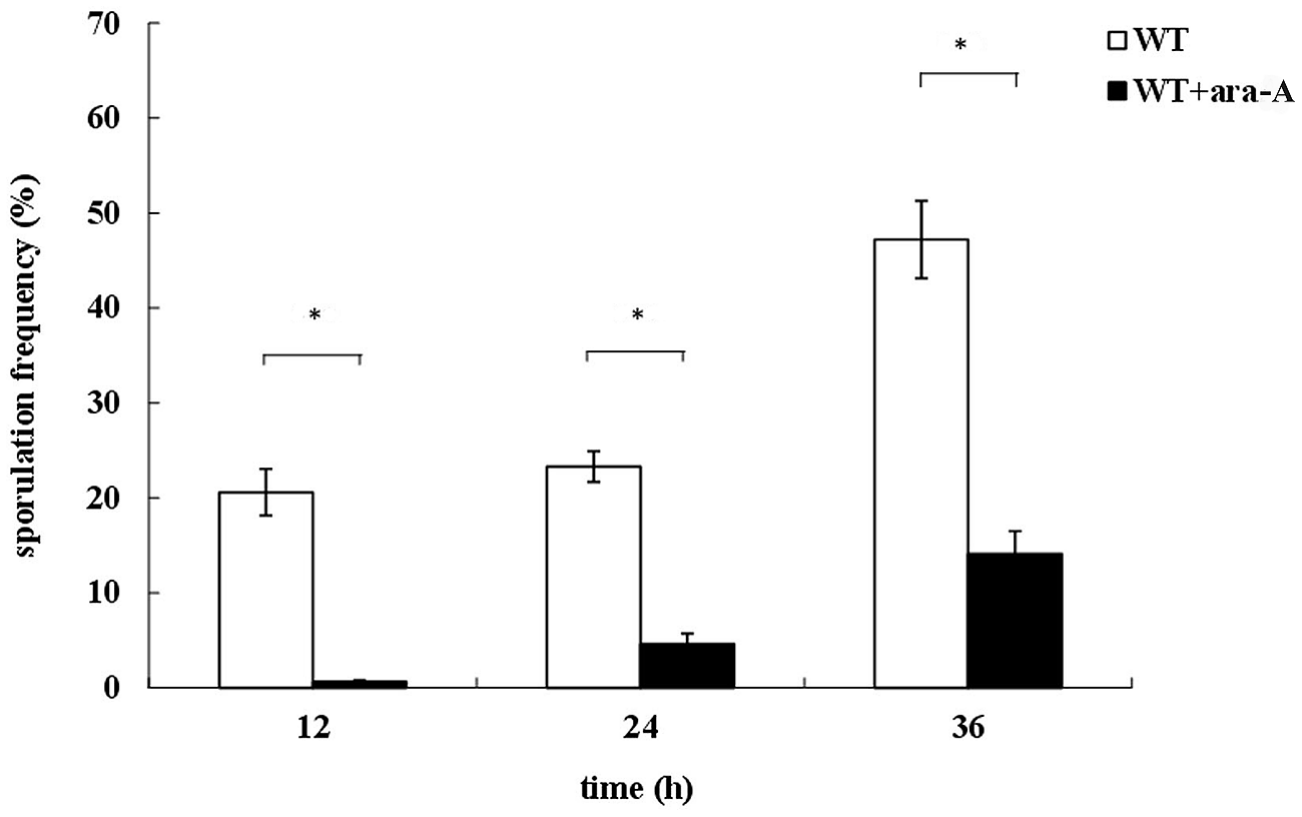
**Defects of sporulation with the treatment of PrkA inhibitor ara-A**. The strain *Bacillus subtilis*168 was cultured in 2x SG medium to induce sporulation. Sporulation frequencies of wild type (WT) strain without ara-A or with addition of ara-A were determined at 12, 24, and 36 h, respectively. The white histogram bar represents the WT strain, and the black bar represents ara-A-treatment group. ^∗^*P* < 0.01.

To determine a possible role of PrkA in *B. subtilis*, the PrkA inhibitor ara-A was added to decrease the activity of PrkA, and then a series of phenotypes, such as vegetative growth, cell morphology, and sporulation were assayed. Our results showed that the addition of ara-A significantly affected the spore numbers. Specifically, statistical analysis revealed that the sporulation frequencies in the ara-A-treatment groups reduced to 0.65 ± 0.14%, 4.61 ± 1.13%, and 14.1 ± 2.42% at 12, 24, or 36 h, respectively, compared to 20.6 ± 2.45%, 23.3 ± 1.60%, and 47.2 ± 4.09% in the negative controls at the same time points (*P* < 0.01; **Figure 1**). Therefore, the significant differences between ara-A-treatment groups and the negative controls suggested that STPK PrkA in *B. subtilis* might be involved in sporulation. However, no obvious differences were observed in vegetative growth and cell morphology between the cultures treated and not treated with PrkA inhibitor ara-A (data not shown).

### Disruption of Gene *prkA* Results in Decreased Sporulation

**FIGURE 2:**
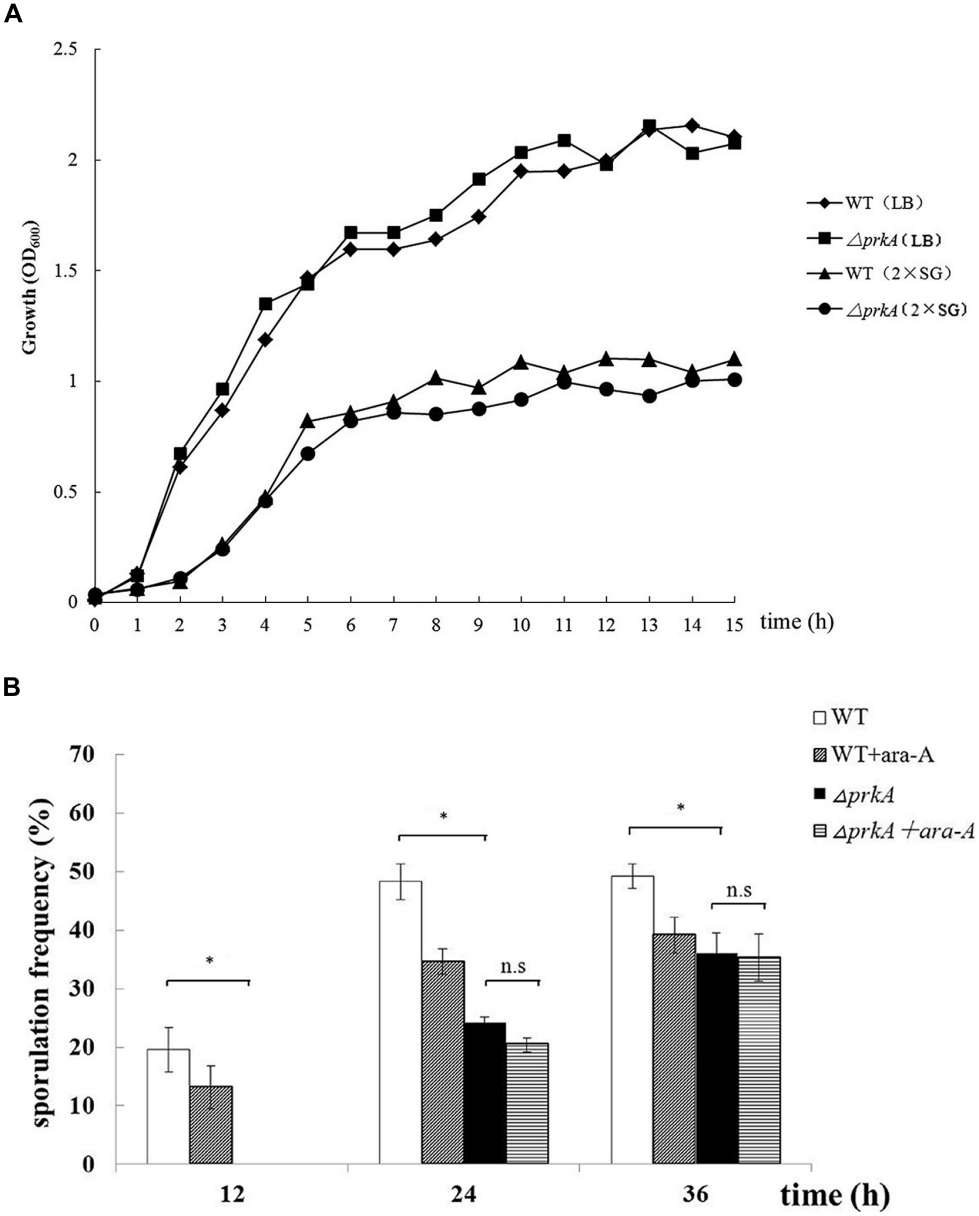
**Disruption of the gene *prkA* decreases sporulation in *B. subtilis* 168. (A)** The vegetative growth rates of Δ*prkA* mutant and *B. subtilis* 168 strain in either LB medium or 2x SG medium. Δ*prkA* mutant (■) and *B. subtilis* 168 in LB medium (◆); Δ*prkA* mutant (•) and *B. subtilis* 168 in 2x SG medium (▲). **(B)** Sporulation frequencies in WT strain *B. subtilis* 168, strain with ara-A, Δ*prkA* mutant strain, and Δ*prkA* mutant with ara-A. ^∗^*P* < 0.01; n.s represented no statistical significance.

To confirm the roles of PrkA in sporulation, the *prkA* deletion strain (PRKA5E) was successfully constructed and its phenotypes were determined. The vegetative growth of Δ*prkA* mutant in either LB medium or 2x SG medium was similar to that of the WT strain (**Figure 2A**), illustrating that the PrkA protein has no effect on the vegetative growth on *B. subtilis*. However, the sporulation frequencies in Δ*prkA* mutant strain decreased significantly with 0%, 24.08 ± 1.16%, and 36.09 ± 3.44%, respectively, at 12, 24 or 36 h; while the WT strain had about 19.63 ± 3.83%, 48.31 ± 3.07%, and 49.26 ± 1.20% of the sporulation frequencies at these time points, respectively, (*P* < 0.01; **Figure 2B**). Compared the two sets of data between the Δ*prkA* mutant and the WT strain, it was also suggested the sporulation was postponed by the deletion of gene *prkA* since no spores were available in Δ*prkA* at the time point of 12 h. Moreover, to determined whether the inhibitor ara-A could affect the sporulation through some other pathways besides inhibition of PrkA, we added the ara-A in the Δ*prkA* mutant strain again. It was found that ara-A had little inhibitory activity in sporulation when the gene *prkA* was knockout, and the sporulation frequencies in Δ*prkA* mutant with the treatment of ara-A were 0%, 20.43 ± 2.61%, 35.28 ± 4.04%, respectively, at 12, 24, 36 h (*P* > 0.05; **Figure 2B**). Our data from the Δ*prkA* mutant confirmed the previous report that PrkA is a sporulation-related protein (Eichenberger et al., 2003).

### PrkA Regulates the Expression of the Transcriptional Factor σ^K^ and its Target Genes

Endospore formation is a very complex multi-stages process, in which many cell type-specific, compartmentalized programs of gene expression are controlled by the cell type-specific activity of RNA polymerase sigma factors (Hilbert and Piggot, 2004). Meanwhile, the promoter region of *prkA* contains a cis-acting element of the spore specific sigma factor σ^E^ and it has also been reported to be transcribed by σ^E^ (Eichenberger et al., 2003; Steil et al., 2005). To define the specific stages that PrkA protein was involved during sporulation, three sporulation-related genes that are controlled by three sigma factors of later stages were selected to construct the reporter plasmids to examine transcription by assaying β-galactosidase activities. Those three sporulation-related genes included *spoIVB* (controlled by σ^F^), *gerD* (controlled by σ^G^), and *gerE* (controlled by σ^K^).

**FIGURE 3:**
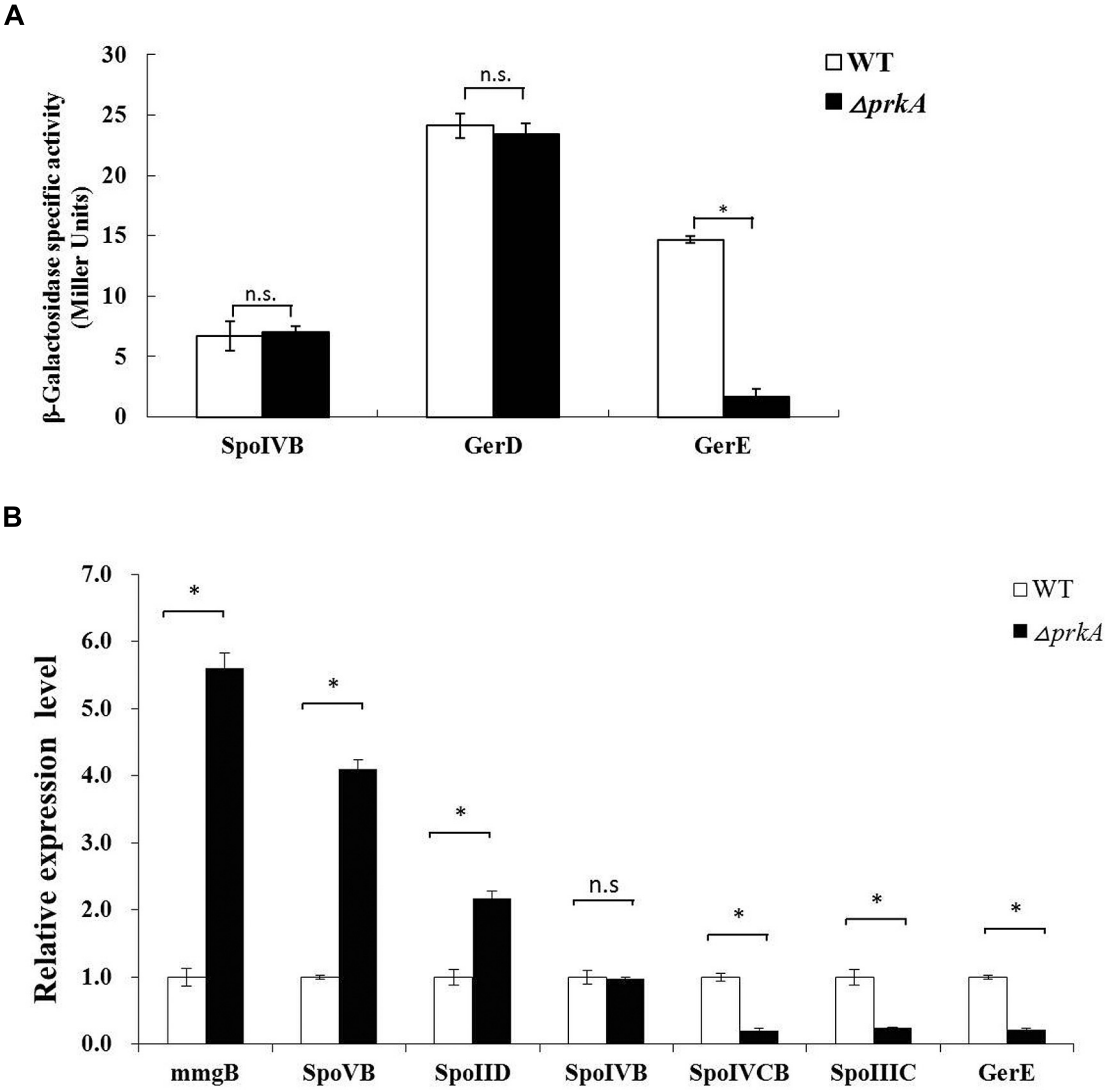
**PrkA decreases the expression of the transcriptional factor σ^K^ and its target genes. (A)** Assay to β-galactosidase activity in the reporter gene fusion expression strains. The white histogram bar represents the WT strain, and the black bar represents Δ*prkA* mutant. **(B)** Real-time PCR to detect the relative expressional level of the typical sporulation-related genes, including *mmgB*, *spoIID*, *spoVB, spoIVB*, *gerD*, and *gerE*. The white histogram bar represents the WT strain *B. subtilis* 168, and the black bar represents Δ*prkA* mutant. ^∗^*P* < 0.01; n.s represented no statistical significance.

The promoter regions of those three genes were cloned and fused to the reporter plasmid pDG1728 to drive the expression of β-galactosidase. Altogether, six strains, including SPOIVBPRKA5E, GERDPRKA5E, GEREPRKA5E as well as the corresponding control strains SPOIVBBS, GERDBS, GEREBS, were successfully constructed by transforming those three recombinant plasmids into the parent strains of Δ*prkA* and *B. subtilis* 168, respectively. After analyzing their β-galactosidase activities, we found that the expression level of gene *gerE*, one of the target genes of the transcriptional factor σ^K^, decreased significantly in the Δ*prkA* mutant strain compared to that in the corresponding control strains (**Figure 3A**).

To further verify our experimental results from the assay of β-galactosidase activity, we compared the expressional levels of *spoIVB*, *sigK*, and *gerE* using real time-PCR between *B. subtilis* 168 and Δ*prkA* mutant strain. At the same time, we also assayed the transcription of those genes *mmgB*, *spoVB*, and *spoIID* that are known to be under the control of σ^E^. The encoding gene for the sigma factor of σ^K^ includes two separate coding regions of *spoIVCB* and *spoIIIC* as mentioned above. It was found that the disruption of gene *prkA* led to decreased expressions of the encoding genes of σ^K^ (*spoIVCB* and *spoIIIC*). The expressional level of *gerE*, which is controlled by σ^K^, showed a reduction similar to the result from β-galactosidase assay. In contrast, compared to the WT strain, the transcriptions of *mmgB*, *spoVB*, and *spoIID* were differentially increased in the Δ*prkA* mutant. The gene *spoIVB* controlled by σ^F^ had the similar mRNA level to WT strain (**Figure 3B**). Thus our results suggested that PrkA was likely involved in regulating the synthesis of transcriptional factor σ^K^, affected the expression of its downstream target genes, and finally caused the deficient sporulation.

### The Complementary Expression of *sigK* Genes Rescued the Defect of Sporulation in Δ*prkA* Mutant Strain

**FIGURE 4:**
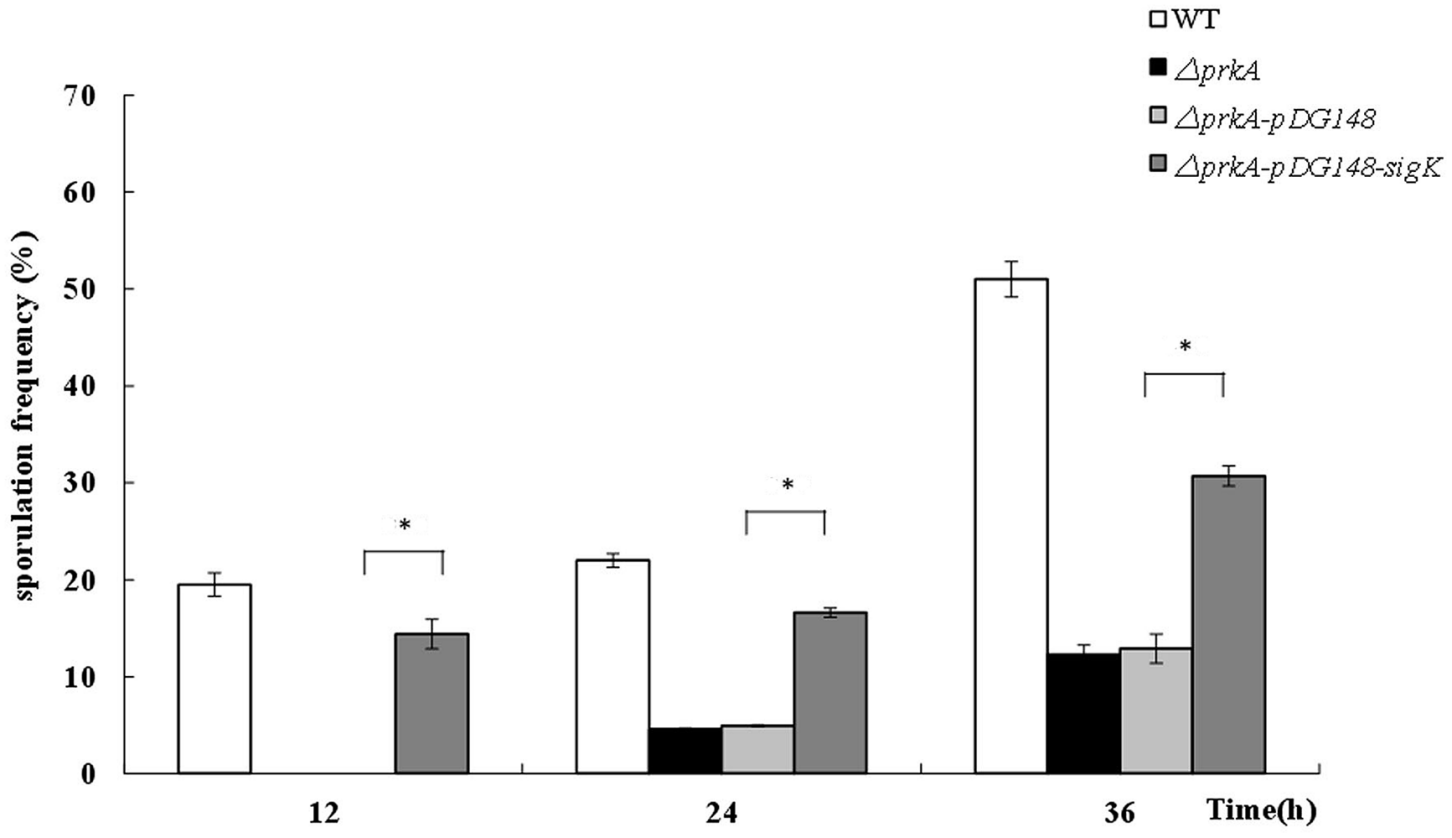
**The complement of *sigK* gene in Δ*prkA* mutant strain partially rescues the effect of sporulation**. Sporulation frequencies were detected in WT strain *B. subtilis* 168, Δ*prkA* mutant, the blank vector pDG148 in Δ*prkA* mutant, and the strain with σ^K^ complement in Δ*prkA* mutant. ^∗^*P* < 0.01.

Based on the above hypothesis that the decreased sporulation level of Δ*prkA* mutant strain was caused by the reduced expressions of the transcriptional factor σ^K^, we designed an experiment to elevate the expression of *sigK* in Δ*prkA* mutant strain. After the recombinant plasmid pSIGKPRKA5E and the blank vector pDG148 were transformed into Δ*prkA* mutant, the spore numbers and sporulation frequencies were determined. Compared to the Δ*prkA* mutant strain, the sporulation of SIGKPRKA5E strain significantly improved to about 60% of sporulation capability in WT *B. subtilis* 168 (**Figure 4**). Thus, the data is consistent with the hypothesis that elevated expression of σ^K^-regulated genes could partially improve the sporulation defect in Δ*prkA* mutant.

### PrkA Positively Controls the Transcriptional Factor σ^K^ Mediated by the Motif of Hpr (ScoC)

**FIGURE 5:**
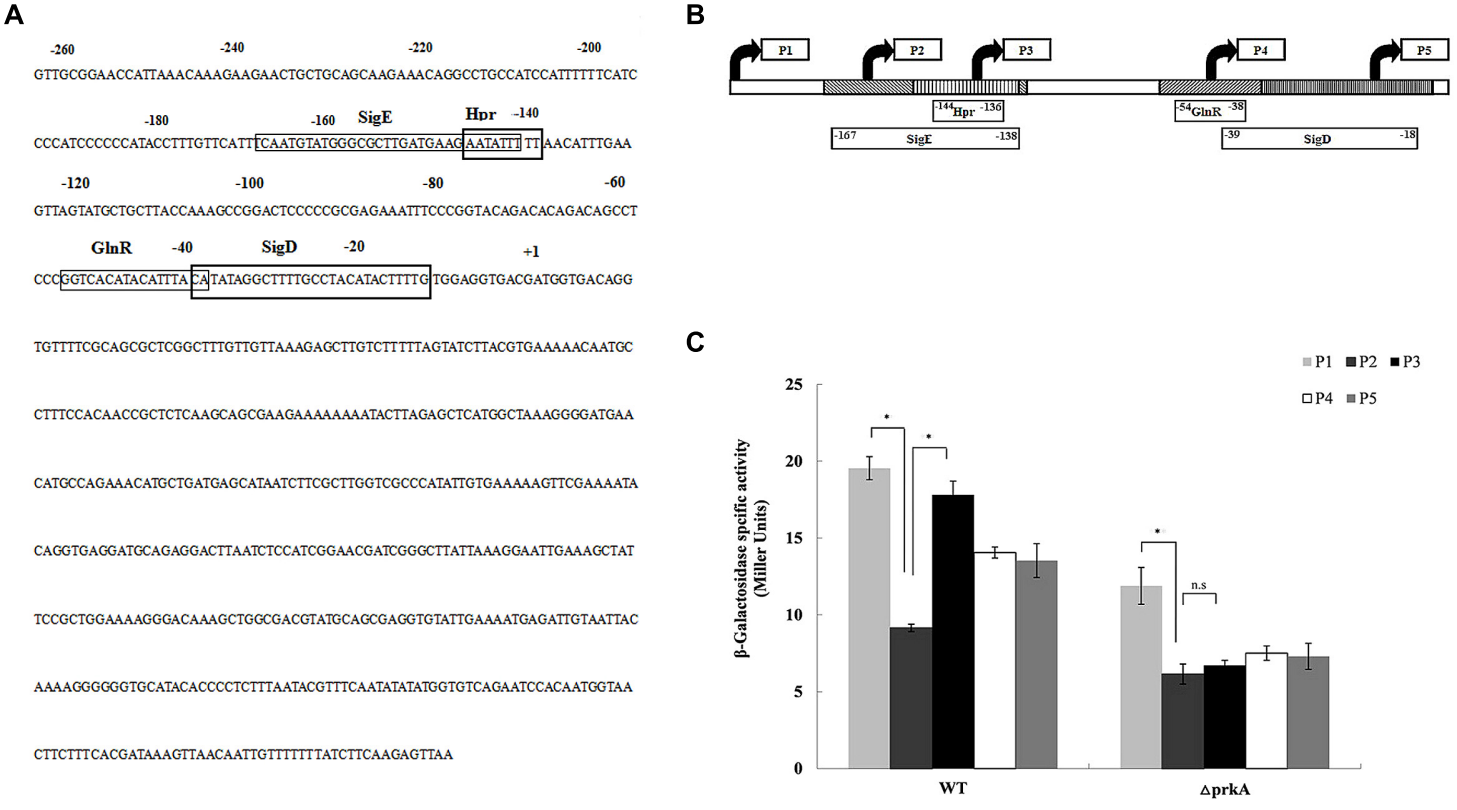
**PrkA changes the expression of *sigK* via negatively regulating the transcription factor of Hpr (ScoC). (A)** The sequence of the *sigK* promoter regions by 5′ deletions. The rectangle boxes indicate the binding motifs in the promoter of *sigK* including SigE, Hpr(ScoC), GlnR, and SigD. **(B)** Mapping of the *sigK* promoter regions by 5′ deletions. Schematic representation of the different *sigK::lacZ* fusions used in this study. The filled arrows indicate the primers used for generating the various reporter fusions (P1–P5). The blocks also indicate the binding motifs in the promoter of *sigK* including SigE, Hpr(ScoC), GlnR, and SigD, whereas the 5′ and 3′ end termini of the motifs are denoted with their nucleotide position relative to the initiator codon. The derivative strains of WT *B. subtilis* 168 or Δ*prkA* mutant carry the respective fusions are shown the below. **(C)** Expression of the series of *sigK*::*lac*Z fusions, respectively. Cells were grown in LB medium at 37°C, and β-galactosidase activities were determined at 36 h. The left histograms represent the derivative strains of the WT background; the right histograms represent the derivative strains of the Δ*prkA* mutant background. ^∗^*P* < 0.01; n.s represented no statistical significance.

To understand how PrkA positively control the expression of the transcriptional factor σ^K^ that is required at the later stage during sporulation, we performed investigation on the regulation of gene expression for the transcriptional factor σ^K^. DBTBS (Database of Transcriptional Regulation in *B. subtilis*) was firstly employed to identify the potential cis-activating elements within the promoter region of *sigK*, and four potential transcription factor binding motifs of SigE, Hpr (ScoC), GlnR, and SigD were found at the 5% significance level. Then, a series of reporter fusions containing the truncated *sigK* promoter regions to *lacZ* were successfully constructed. Specifically, five nested fragments with a common downstream end and variable upstream ends were fused to promoter-less *lacZ* gene in pDG1728, respectively (**Figure 5A,B**). Those constructed plasmids were transformed into the Δ*prkA* mutant strain and the WT *B. subtilis* 168. The β-galactosidase activity in each strain was measured.

In the five strains containing the reporter plasmids transformed into the WT *B. subtilis* 168, we found that strain WT-P2 had an obviously lower β-galactosidase activity than WT-P1 (*P* < 0.01). Strain WT-P1 contained an additional transcriptional motif of SigE than strain WT-P2. However, WT-P2 had much lower β-galactosidase activity than WT-P3 that missed the cis-activating element of Hpr (ScoC; *P* < 0.01; **Figure 5C**). The β-galactosidase activities in WT-P4 and WT-P5 had no statistical differences (*P*> 0.05). Based on the experimental data, we confirmed that, in WT strain *B. subtilis* 168, the expression of *sigK* was under the transcription factor of SigE that had been described previously (Kroos et al., 1989). However, it was firstly shown in our study that the transcription factor Hpr (ScoC) negatively regulated the expression of *sigK*.

In the analysis of strains with plasmids transformed into Δ*prkA* mutant, the β-galactosidase activity in prkA-P2 was much lower than in prkA-P1 (*P* < 0.01), while prkA-P1 was similar to that in the corresponding strain transformed from WT *B. subtilis* 168. However, no significant difference was observed between prkA-P2 and prkA-P3 (*P*> 0.05; **Figure 5C,B**). Since the reporter plasmids prkA-P2 had an additional binding motif of Hpr (ScoC) that potentially controlled *sigK* expression negatively in the WT strain, the absence of PrkA inhibited Hpr (ScoC) to regulate the expression of *sigK* anymore, suggesting that PrkA increased the expression of *sigK* via the transcription factor binding motif of Hpr (ScoC).

### Hpr (ScoC) Functions Epistatic to PrkA in the Spore Development

**FIGURE 6:**
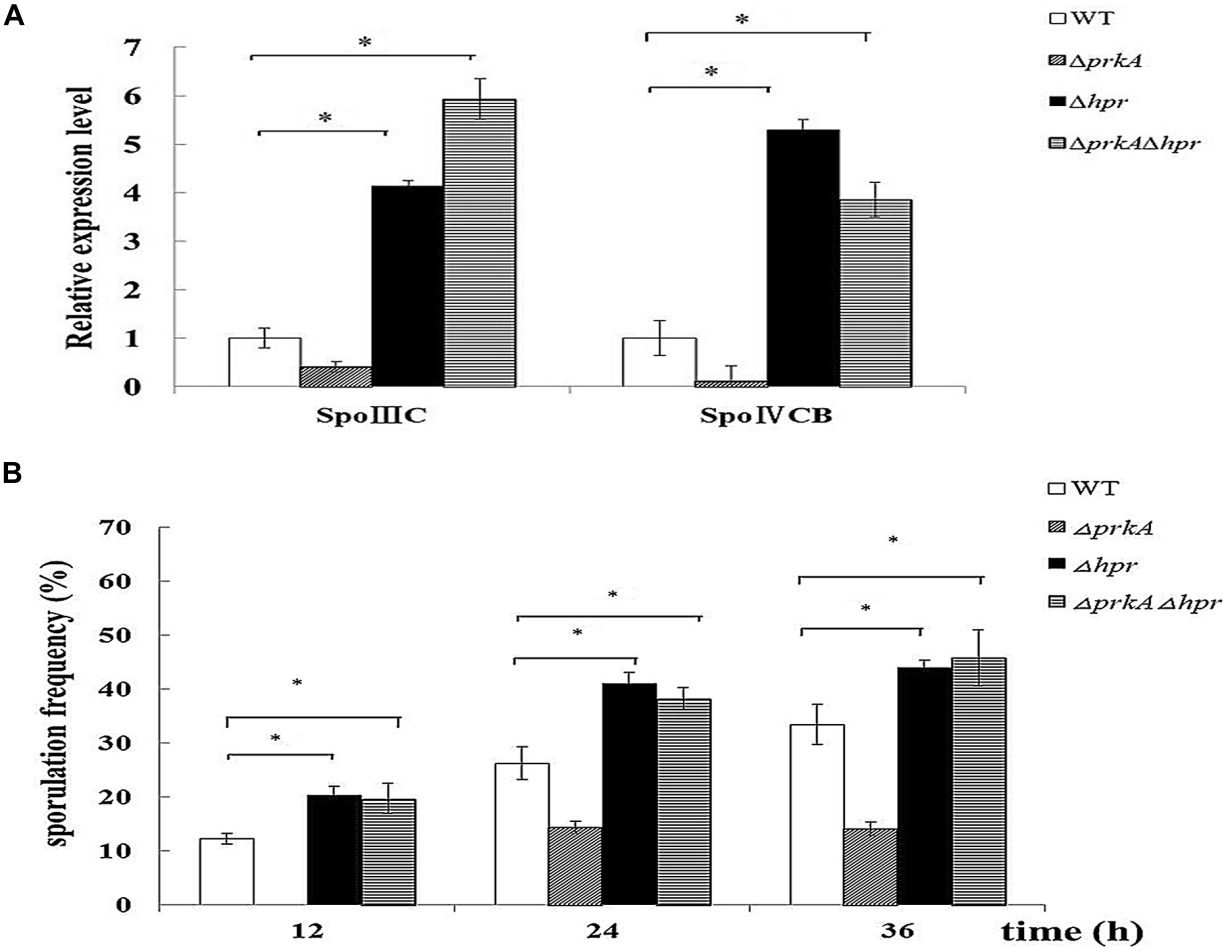
**Hpr (ScoC) negatively regulates the expression of *sig*K in *B. subtilis* 168, and functions epistatic to PrkA in sporulation. (A)** Hpr (ScoC) increases the expression of the transcriptional factor σ^K^. Real-time PCR was employed to detect the relative expression levels of the transcriptional factor σ^K^, which included spoIIIC and spoIVCB. **(B)** Effect of the gene *hpr* on sporulation. Sporulation frequencies in WT strain *B. subtilis* 168, Δ*prkA* mutant, Δ*hpr* mutant, and Δ*prkA*Δ*hpr* mutant were determined at 12, 24, 36 h, respectively. ^∗^*P* < 0.01.

It was been shown in our β-galactosidase assay that the motif Hpr (ScoC) negatively regulated the expression of *sig*K in the WT *B. subtilis* 168. To further confirm such a conclusion, we assessed if the mRNA levels of *spoIIIC* and *spoIVCB* were influenced by the gene *hpr*. In Δ*hpr mutant,* the mRNA levels of both *spoIIIC* and *spoIVCB* increased about 4.5-fold compared to the WT *B. subtilis* 168 (*P* < 0.01; **Figure 6A**), suggesting that Hpr indeed negatively controls the expression of *sigK*. Moreover, we also constructed the double mutant Δ*prkA*Δ*hpr* and detected the expression of *sigK* again. It was shown that Δ*prkA*Δ*hpr* had the similarly higher mRNA levels than the WT (*P* < 0.01; **Figure 6A**).

Then, we examined whether Hpr functioned in the spore development. Our result showed that the sporulation frequencies in Δ*hpr* strain were 20.22 ± 2.02%, 41.04 ± 2.11%, 43.97 ± 0.90% at 12, 24, 36 h, respectively, which were obviously higher than that of the WT strain (12.25 ± 1.01%, 26.24 ± 3.00%, and 33.43 ± 3.72% at these time points; *P* < 0.01; **Figure 6B**). Likewise, the double mutant Δ*prkA*Δ*hpr* had the comparable sporulation frequencies as Δ*hpr* mutant (*P*> 0.05; **Figure 6B**). Together, these experiments confirmed that Hpr (ScoC) negatively regulated the expression of *sig*K in *B. subtilis* 168, and functioned epistatic to PrkA in the spore development.

## Discussion

Under starvation or other environmental stress, Gram-positive bacterium initiates a series of sporulation-related events to generate dormant endospores. The endospores can survive treatments that would otherwise rapidly and efficiently kill other bacterial forms. Such conditions include high temperatures, ionizing radiation, chemical solvents, detergents, and hydrolytic enzymes (Nicholson et al., 2000). Thus, the abilities to form tough, resistant endospores in bacteria have become an important factor in influencing the pathogenesis in pathogenic bacterium or agricultural application in biocontrol agents (Nicholson, 2002; Spencer, 2003). Furthermore, the process of sporulation involves in an unusually mechanism of asymmetric cell division, and attracts much attentions in biological research (Harry, 2001; Ben-Yehuda and Losick, 2002). As a kind of typical pattern of microorganisms, the best-studied paradigm of spore-forming is *B. subtilis*.

During sporulation in *B. subtilis*, a strict program of sigma factors activation, including σ^H^, σ^F^, σ^E^, σ^G^, and σ^K^, has been demonstrated as one of the most important events to ensure that sporulation gene expressions occur at the right time and place (Driks, 1999; Errington, 2003). The cascade of those four sigma factors (σ^F^, σ^E^, σ^G^, and σ^K^) is triggered in a sequential order and their biological functions are involved in the engulfment of the smaller cell by the larger sibling, the formation of forespore and the mother cell, and final the assembly of spore coat (Piggot and Hilbert, 2004). Their roles are mainly compartment specific, but also interactional. The sigma factors σ^F^ and σ^G^ function in the forespores, where the transcription of *spoIIIG* gene (encoding σ^G^) depends on the RNA polymerase containing σ^F^-subunit. Meanwhile, it has also been suggested that the σ^E^-directed signals from the mother cell are necessary for the formation and activation of σ^G^ (Piggot and Hilbert, 2004). The activities of σ^E^ and σ^K^ are confined within the mother cells, where the synthesis of σ^K^ is under the control of σ^E^. But after translation, σ^K^ will remain as an inactive precursor in the mother cell until its activation by a proteolysis event, in which the σ^G^ -controlled gene *spoIVB* plays an important role (Wakeley et al., 2000; Campo and Rudner, 2007). Because sporulation itself is a complex multi-gene regulation process, a gene regulatory network should exist to accurately control the expression of sigma factor.

**FIGURE 7:**
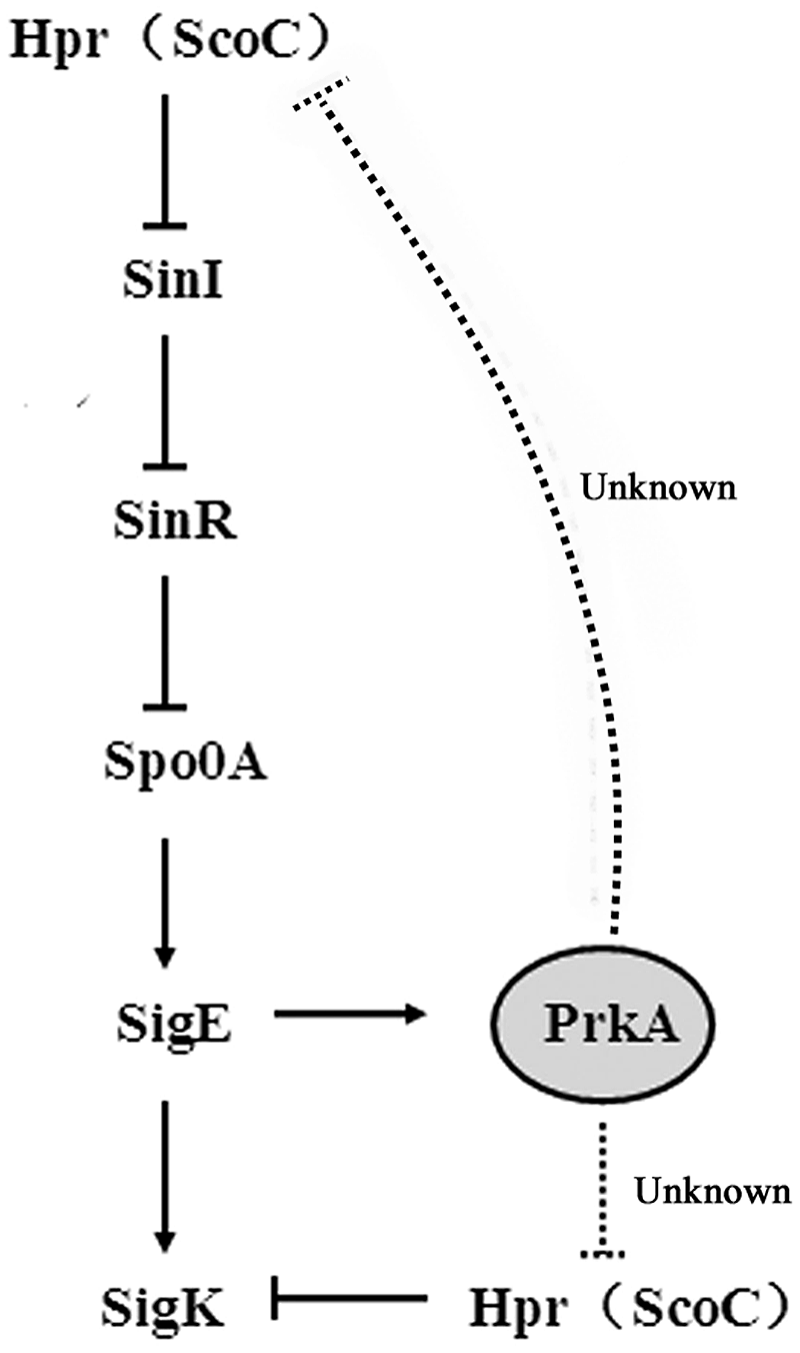
**A proposed model of PrkA regulating the expression of *sigK* in *B. subtilis* 168**.

Our current investigation suggested that though the expression of σ^K^ was controlled by the well-known sigma factor of σ^E^, PrkA can also negatively regulated Hpr (ScoC) to induce the expression of *sigK*. In mammalian cells, STPK PrkA belongs to AMPK superfamily, and acts as a master regulator of the main energy metabolism pathway. However, few investigations of PrkA have been reported in prokaryotic cells and thus its function requires further elucidated. Our results here demonstrated that the absence of PrkA significantly reduced the sporulation in *B. subtilis* 168 that is also consistent with the results previously reported (Eichenberger et al., 2003). The addition of PrkA inhibitor ara-A or the deletion of the gene *prkA* both showed a similar pattern, confirming the biological function of PrkA as a sporulation-related protein. To analyze the specific stages of sporulation that PrkA influences, the expressional levels of the important sigma factors as well as their downstream genes were assayed using reporter gene of *lacZ* and qPCR. Our data further suggested that the decreased expression of the transcriptional factor σ^K^ and its downstream genes caused by the absence of PrkA were the main reason of reduced sporulation. This hypothesis was confirmed by the complementary expression of *sigK* in Δ*prkA* mutant. Combining the results from bioinformatics analysis and reporter gene activity assay, our study revealed that PrkA controls the expression of σ^K^ via the transcription factor binding motifs of Hpr (ScoC). Meanwhile, we have also noticed an indirect pathway described in previous literature: Hpr (ScoC) influencing σ^E^ expression by negatively regulating SinI, SinR, and Spo0A (Koide et al., 1999; Kodgire and Rao, 2009), and ultimately decreasing σ^K^ (**Figure 7**). In our transcriptional analysis, it consistently illustrated that prkA-P1 strain (Δ*prkA* mutant with the reporter fusion containing SigE binding motif) had lower β-galactosidase activity than that WT-P1 (WT strain with the reporter fusion containing SigE binding motif; **Figure 5**).

## Conclusion

Our current investigation confirms the biological role of PrkA in sporulation. In addition, we revealed the underlying mechanism of how PrkA influenced the expression of sigma factor σ^K^ by the negative regulation of Hpr (ScoC).

## Conflict of Interest Statement

The authors declare that the research was conducted in the absence of any commercial or financial relationships that could be construed as a potential conflict of interest.
